# Distinct HLA class II alleles influence antibody response to the *Plasmodium vivax* Duffy binding protein

**DOI:** 10.1186/1475-2875-13-S1-P51

**Published:** 2014-09-22

**Authors:** Flora S Kano, Flávia A Souza-Silva, Taís N Sousa, Jéssica RS Alves, Michaelis L Tang, Roberto S Rocha, Cristiana FA Brito, Ana Maria Sell, Luzia H Carvalho

**Affiliations:** 1 Centro de Pesquisas René Rachou/FIOCRUZ, Belo Horizonte, MG, Brazil; 2 Centro de Pesquisas Leonidas e Maria Deane/FIOCRUZ, Manaus, AM, Brazil; 3 Universidade Estadual de Maringá, Maringá, Pr, Brazil

## 

*Plasmodium vivax* infects human reticulocytes through a major pathway that requires interaction between an apical parasite protein, the Duffy binding protein (region II, DBPII) and its cognate receptor on reticulocytes, the Duffy antigen receptor for chemokines (DARC). Although most people naturally exposed to *P. vivax* fail to develop antibodies that inhibit the DBPII-DARC interaction, the genetic factors that modulate humoral responsiveness are poorly characterized. Aiming to investigate if DBPII non-responsiveness could be HLA class II-linked (DRB1, DQB1 and DQA1), we carried-out an open cohort study among 340 non-related volunteers in an agricultural settlement of the Brazilian Amazon; three cross-sectional surveys were conducted at 6-month intervals, and 240 out of 340 (71%) subjects had consecutive samples. At enrolment, 40.9% of the study population had DBPII IgG antibodies, as measured by conventional serology (ELISA); two alleles at HLA-DR locus (DRB1*10:01 and DRB1*14:02) were negatively associated with the antibody response, while two alleles at HLA-DQ locus (DQB1*02:02 and DQA1*01:03) were positively associated with antibody response. The 12-month follow-up study confirmed that those alleles associated with positive antibody response were also associated with the persistence of this specific antibody response. Further, we investigated whether HLA class II polymorphisms would influence the functional proprieties of DBPII antibodies (BIAbs, binding inhibitory antibodies), as assessed by the COS-7 cytoadherence assay. The results demonstrated that four alleles were associated with the presence (DRB1*07:01; DQB1*02:02, DQA1*02:01) or absence (DRB1*16:02) of DBPII BIAbs response. Taken together, this is the first demonstration that HLA class II polymorphisms might influence the frequency and stability of the DBPII immune response. Due to the relevance of these findings for vaccines now in development, it would be pertinent to investigate whether such an association exists in other *P. vivax* malaria endemic areas.

